# Supplementary material for: C→U transition biases in SARS-CoV-2: still rampant 4 years from the start of the COVID-19 pandemic
Source: mBio. 2024 Oct 30;15(12):e02493-24. doi: 10.1128/mbio.02493-24 (PMC11633203; doi:10.1128/mbio.02493-24)
Supplement: Supplemental material — Table S2 and Fig. S1. [file mbio.02493-24-s0001.docx]

SUPPLEMENTARY DATA

TABLE S2

EXCESS SUBSTITUTIONS OVER EXPECTED NUMBERS BASED ON POISSON DISTRIBUTION^1^

A) 5’ (upstream) base context:

|  |  | **Lineage totals** | | | | | | | | | |
| --- | --- | --- | --- | --- | --- | --- | --- | --- | --- | --- | --- |
| **Substitution** | **RNA** | **1** | **2** | **3** | **4** | **5** | **6** | **7** | **8** | **9** | **10** |
| A->G | 5’U | 18 | 218 | 137 | 66 | 50 | 48 | 7 | 0 | 0 | 0 |
|  | 5’A/C/G | 730 | 773 | 357 | 140 | 25 | 6 | 7 | 16 | 18 | 0 |
| G->A | 5’U | 186 | 296 | 158 | 44 | 20 | 0 | 7 | 0 | 0 | 0 |
|  | 5’A/C/G | -105 | 409 | 466 | 244 | 134 | 36 | 7 | 8 | 9 | 0 |
| U->C | 5’U | 51 | 225 | 91 | 41 | 10 | 6 | 0 | 8 | 0 | 0 |
|  | 5’A/C/G | 392 | 984 | 584 | 306 | 139 | 54 | 35 | 0 | 0 | 0 |
| C->U | 5’U | -718 | -791 | -573 | -225 | 135 | 246 | 518 | 398 | 429 | 169 |
|  | 5’A/C/G | -1289 | -1333 | -588 | 201 | 598 | 1018 | 1091 | 1115 | 687 | 89 |
| G->U | 5’U | -59 | 201 | 289 | 227 | 189 | 96 | 49 | 0 | 9 | 10 |
|  | 5’A/C/G | -420 | 152 | 321 | 336 | 233 | 174 | 98 | 56 | 9 | 0 |

B) RNA binding

|  |  | **Lineage totals** | | | | | | | | | |
| --- | --- | --- | --- | --- | --- | --- | --- | --- | --- | --- | --- |
| **Substitution** | **RNA** | **1** | **2** | **3** | **4** | **5** | **6** | **7** | **8** | **9** | **10** |
| A->G | Paired | 348 | 469 | 284 | 117 | 45 | 36 | 7 | 8 | 9 | 0 |
|  | Unpaired | 400 | 524 | 209 | 93 | 30 | 18 | 7 | 8 | 9 | 0 |
| G->A | Paired | 59 | 507 | 457 | 203 | 114 | 24 | 0 | 0 | 9 | 0 |
|  | Unpaired | 23 | 198 | 170 | 85 | 40 | 12 | 14 | 8 | 0 | 0 |
| U->C | Paired | 207 | 548 | 343 | 171 | 69 | 24 | 7 | 8 | 0 | 0 |
|  | Unpaired | 236 | 661 | 332 | 176 | 80 | 36 | 28 | 0 | 0 | 0 |
| C->U | Paired | -1112 | -1141 | -564 | -1 | 331 | 321 | 404 | 357 | 157 | 19 |
|  | Unpaired | -895 | -983 | -597 | -23 | 402 | 943 | 1205 | 1156 | 960 | 239 |
| G->U | Paired | -325 | 265 | 382 | 384 | 203 | 150 | 49 | 32 | 9 | 0 |
|  | Unpaired | -153 | 88 | 229 | 179 | 224 | 120 | 98 | 24 | 9 | 10 |

^1^Observed substitution totals in in each variability category – predicted number based on a random (Poisson) distribution.

FIGURE S1


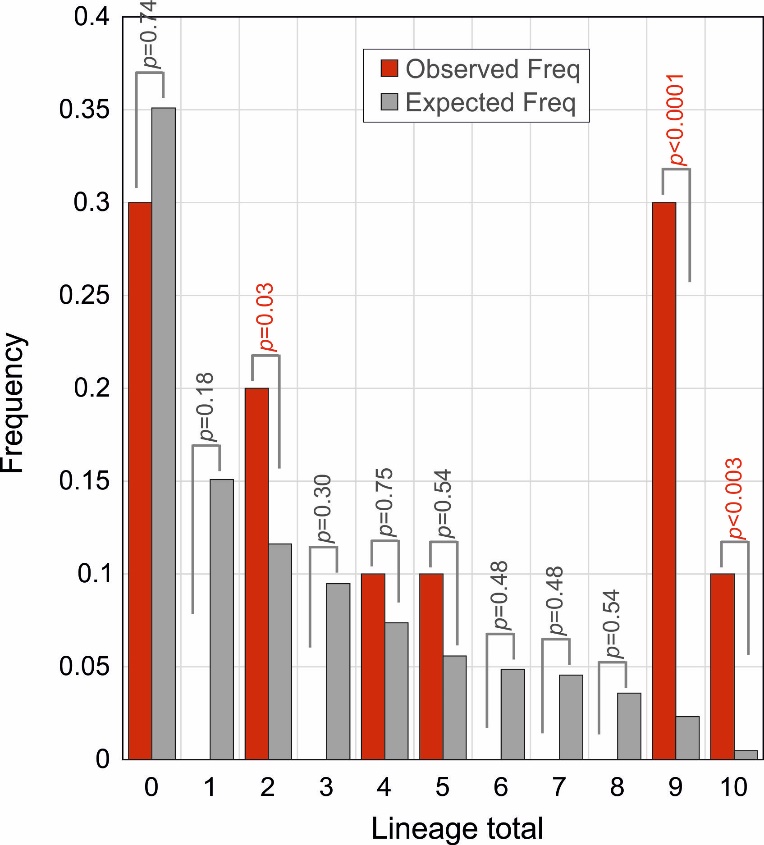


The distribution of C-U transitions at sites of previously reported *in vitro* A3A-induced mutations (18) compared to the observed distribution of unfixed C->U changes; differences in lineage distributions with observed values were calculated by Pearson chi-square.
